# Preventive Activity of an Arginine-Based Surfactant on the Formation of Mixed Biofilms of Fluconazole-Resistant *Candida albicans* and Extended-Spectrum-Beta-Lactamase-Producing *Escherichia coli* on Central Venous Catheters

**DOI:** 10.3390/antibiotics14030227

**Published:** 2025-02-24

**Authors:** Lourdes Pérez, Cecília Rocha da Silva, Lívia Gurgel do Amaral Valente Sá, João Batista de Andrade Neto, Vitória Pessoa de Farias Cabral, Daniel Sampaio Rodrigues, Lara Elloyse Almeida Moreira, Maria Janielly Castelo Branco Silveira, Thais Lima Ferreira, Anderson Ramos da Silva, Bruno Coêlho Cavalcanti, Nágila Maria Pontes Silva Ricardo, Francisco Alessandro Marinho Rodrigues, Hélio Vitoriano Nobre Júnior

**Affiliations:** 1 Department of Surfactants and Nanobiotechnology, Instituto de Química Avanzada de Cataluña, Consejo Superior de Investigaciones Químicas (IQAC-CSIC), 08034 Barcelona, Spain; anderson_ramos3@hotmail.com; 2 Laboratory of Bioprospection in Antimicrobial Molecules (LABIMAN), School of Pharmacy, Federal University of Ceará, Fortaleza 60020-181, Brazil; cecilia@ufc.br (C.R.d.S.); liviagurgel@ufc.br (L.G.d.A.V.S.); joao.neto@unichristus.edu.br (J.B.d.A.N.); vitoriaffarias15@gmail.com (V.P.d.F.C.); dsampaior@gmail.com (D.S.R.); laraeamoreira@gmail.com (L.E.A.M.); janiellycastelobranco@gmail.com (M.J.C.B.S.); thaislimaferreira11@gmail.com (T.L.F.); 3 Center of Drug Research and Development, Federal University of Ceará, Fortaleza 60020-181, Brazil; bccavalcanti@gmail.com; 4 Christus University Center (UNICHRISTUS), Fortaleza 60160-230, Brazil; 5 Polymer and Materials Innovation Laboratory (LABPIM), Department of Organic and Inorganic Chemistry, Sciences Center, Federal University of Ceará, Fortaleza 60440-900, Brazil; naricard@ufc.br (N.M.P.S.R.); alessandroquimica@gmail.com (F.A.M.R.)

**Keywords:** *Candida albicans*, *Escherichia coli*, biofilm, surfactant, central venous catheters

## Abstract

**Background/Objectives:** Mixed bloodstream infections associated with central venous catheter (CVC) use are a growing problem. The aim of this study was to evaluate the activity of a cationic arginine-based gemini surfactant, C_9_(LA)_2_, against mixed biofilms of fluconazole-resistant *Candida albicans* and extended-spectrum beta-lactamase (ESBL)-producing *E. coli*, and the preventive effect of this surfactant impregnated in CVCs on the formation of inter-kingdom biofilms. **Methods:** Broth microdilution assays were performed along with evaluation of the effect against mixed biofilms in formation. The impregnation of CVCs with the surfactant and with a hydrogel containing the cationic surfactant was investigated to assess their potential to prevent the formation of mixed biofilms. Scanning electron microscopy (SEM) was also utilized. **Results:** Minimum inhibitory concentrations (MICs) for resistant *C. albicans* ranged from 4–5.3 µg/mL, while for *E. coli*, the MICs varied from 85.3 to 298.7 µg/mL. Fungicidal and bactericidal action patterns were obtained. In mixed biofilm formation in 96-well plates, there was a significant reduction in the colony-forming unit (CFU) count. The impregnation of the CVC with C_9_(LA)_2_ alone resulted in a biofilm reduction of 62% versus *C. albicans* and 48.7% against *E. coli* in terms of CFUs. When the CVC was impregnated with the surfactant hydrogel, the effect was improved with an inhibition of 71.7% for *C. albicans* and 86.7% for *E. coli*. The images obtained by SEM corroborated the results. **Conclusions:** C_9_(LA)_2_ has potential for use in CVC impregnation to prevent the formation of mixed biofilms of fluconazole-resistant *C. albicans* and ESBL-producing *E. coli*.

## 1. Introduction

Biofilms are defined as microbial communities attached to a biotic or abiotic surface and surrounded by an extracellular polymeric matrix [1]. It can be formed by single or multispecies communities, as well as between different kingdoms [2]. In particular, studies have shown that the opportunistic pathogenic yeast *Candida albicans* can form polymicrobial biofilms together with a variety of bacteria, both in vitro and in vivo [3].

The interactions of different microorganisms can influence the expression of virulence factors, the host’s immune response and the effectiveness of antimicrobial drugs, as well as the outcome of infections [3]. For example, Farrokhi et al. [4] investigated the in vitro interaction of *Escherichia coli* and *C. albicans* mixed biofilms, and it was found that biofilm formation increased 2.2 times in the presence of *E. coli*, and *Candida albicans* dissemination from the mixed biofilm increased by up to 2.7 times.

Among the most common infections generated by microbial biofilms, those associated with intravascular catheters become a concern, leading to bloodstream infections and increasing mortality and costs related to medical care [1]. *C. albicans* is one of the main pathogens associated with catheter-related bloodstream infections (CRBSIs), and about 30–60% of *C. albicans* infections are reported to occur simultaneously with other pathogens. Additionally, *E. coli* has gradually surpassed Gram-positive bacteria as the predominant source of CRBSIs [5,6].

Zhong et al. [7] reported that 26.3% of candidemia in a Chinese hospital was associated with mixed bloodstream infections caused by *Candida* species and bacteria, and 32.1% of those were associated with the use of a central venous catheter (CVC), with Gram-negative bacteria being responsible for 61.2% of cases.

Studies have shown that impregnating CVCs with antimicrobial agents can prevent the formation of biofilms in the early stage (during microbial adhesion), leading to a reduction of infections associated with their use [8,9,10].

Catheters coated/impregnated with chlorhexidine/silver sulfadiazine and antimicrobials are already available on the market, and these medical devices were related to a great reduction of the incidence of catheter colonization, in addition to a decrease in the incidence of CRBSIs per 1000 catheter days [11]. Considering that CVCs have been shown to carry a 64-fold higher risk of resulting in CRBSI compared to peripheral venous catheters, this strategy can improve patient prognoses [12].

In order to mitigate infections associated with catheter use, hydrophilic-coated catheters such as hydrogels loaded with drugs have been described. Hydrogel materials are biocompatible and have been shown to effectively prevent microbial fixation and reduce cell adhesion and biofilm formation on catheters [13]. According to this, the possibility of applying gels loaded with surfactants for impregnation in catheters stands out as an alternative to reduce the risk of biofilm formation in medical devices and CRBSIs [14].

Gemini surfactants are potential substances for the development of new antimicrobial strategies, as exemplified by the cationic arginine-based surfactants described by Sousa et al. [15]. Gemini surfactants containing amino acids, such as arginine, provide a combination of advantages in terms of efficiency, biocompatibility, biodegradability and aquatic toxicity. Given these aspects, in the present work, we used a gemini surfactant from arginine (C_9_(LA)_2_) for application in CVCs.

Currently, it is well known that the emergence of resistant pathogens poses a major threat to human health. Previous studies evaluated the antimicrobial activity of C_9_(LA)_2_ against a variety of sensitive ATCC bacteria and its effect against the biofilm of *Pseudomonas aeruginosa* and methicillin-resistant *Staphylococcus aureus* isolates was demonstrated [15,16]. Considering their epidemiological relevance, in this work, we extended the comprehension of the antimicrobial activity of C_9_(LA)_2_ against two resistant pathogens (fluconazole-resistant *C. albicans* and extended-spectrum-beta-lactamase-producing *E. coli*), both isolated and associated with mixed biofilms. Moreover, we have evaluated the preventive effect of the surfactant alone and the surfactant loaded in a hydrogel against mixed biofilms of those strains in CVCs. This is the first time that we or other authors have used this gemini surfactant to conduct such research.

## 2. Results and Discussion

### 2.1. C_9_(LA)_2_ Chemical Characterization

**Figure 1:**
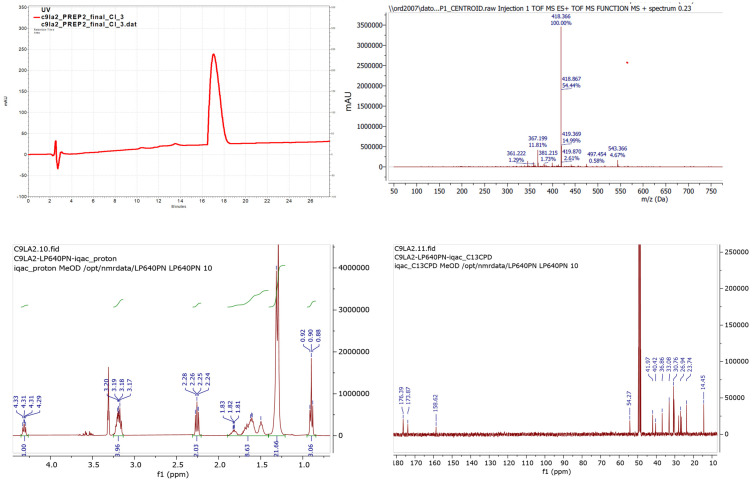
HPLC, mass spectra, ^1^HNMR and ^13^CNMR corresponding to C_9_(LA)_2_.

**Scheme 1:**
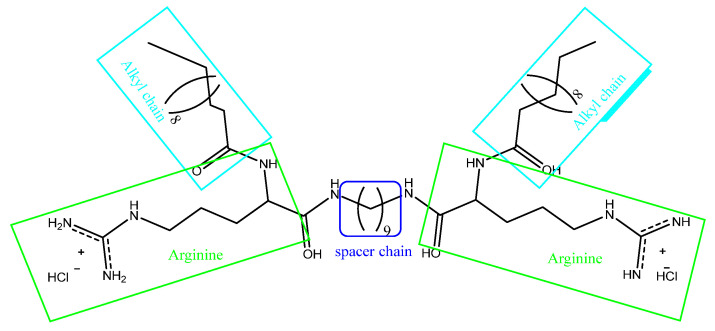
Chemical structure of C_9_(LA)_2_. Green boxes (arginine amino acid), blue boxes (hydrophobic chains) and purple box (spacer chain).

The surfactant C_9_(LA)_2_ was prepared according to Pérez et al. [17]. The NMR spectroscopy, mass spectroscopy and HPLC characterization results (Figure 1) agree with those reported by the referenced study [17]. C_9_(LA)_2_ consists of two monomeric arginine-based amphiphiles linked by a spacer of nine CH_2_ groups at the level of their head (Scheme 1). This compound was very effective in reducing the surface tension, with a very low critical micellar concentration (CMC) value (2.8 µM), and it exhibited interesting antimicrobial and antifungal activities [16]. Moreover, it forms viscous aqueous solutions that contain large aggregates like helicoidal or worm-like micelles [18].

### 2.2. C_9_(LA)_2_ Antimicrobial Activity Against Fluconazole-Resistant C. albicans and ESBL-Producing E. coli

The rising prevalence of resistant bacteria and fungi has increased the demand for the development of novel antimicrobial and antibiofilm compounds. Years ago, the antibacterial and antifungal activity of C_9_(LA)_2_ was evaluated using American Type Culture Collection (ATCC) reference bacteria and fungi [16]. In this work, we describe the measurement of its minimum inhibitory concentration (MIC) values against planktonic cells of some fluconazole-resistant *C. albicans* isolates and third-generation cephalosporin-resistant Enterobacterales (ESBL) *E. coli* strains. These two microbes are highlighted by the World Health Organization as critical priority pathogens to guide research, development and public health actions to control and prevent antimicrobial resistance [19,20].

Regarding planktonic cells, C_9_(LA)_2_ exhibited very good activity against *C. albicans* strains, and MICs varied between 4–5.3 µg/for this species (Table 1). The MICs for *E. coli* isolates ranged from 85.3 to 298.7 µg/mL (Table 2). The previous study reported a MIC value of 128 µg/mL for *C. albicans* ATCC 10231 and >128 µg/mL for *E. coli* ATCC 27325 [16]. The differences can be attributed to the variety of strains and media used to determine the MIC values. Interestingly, the activity of C_9_(LA)_2_ against the ATCC reference strains (*C. parapsilosis* ATCC 22019, *C. krusei* ATCC 6258, *E. coli* ATCC 8739 and *E. coli* ATCC 25922) were similar to that found against fluconazole-resistant *C. albicans* strains and ESBL-producing *E. coli* (Table 1 and Table 2). This behavior can be ascribed to the mode of action of this compound.

The interaction of cationic surfactants with bacteria and fungi involves two steps: (a) attachment to the biological membrane via electrostatic interactions between the cationic charge of the polar head and the negatively charged molecules in the bacterial/fungal membranes; and (b) hydrophobic interactions between the alkyl chain of surfactants and the lipid bilayers of membranes [21]. The anionic components in the inner membrane are around 26% for *E. coli* [22], and the outer membrane leaflet of *E. coli* is dominated by lipopolysaccharides (LPSs) that show multiple negative charges [23]. These LPSs may effectively mop up oppositely charged and hydrophobic molecules [21], causing higher MICs against bacteria than *Candida* strains. Note that the cell membrane is an interesting target to consider when developing new antimicrobial drugs, given that it is complicated for microbes to generate resistance against this type of mechanism [24]. In this regard, studies showed that the mechanism of action of different cationic surfactants from arginine against *C. albicans* and *E. coli* involves alterations of cell membrane permeability and mitochondrial damage [18,25]. Thus, C_9_(LA)_2_ probably has a similar mode of action.

The minimum fungicidal concentration (MFC) and minimum bactericidal concentration (MBC) values were similar to the MICs. The MFC/MIC ratio was between 1.0 and 1.7 and the MBC/MIC ratio was between 1.0 and 1.2. Thus, C_9_(LA)_2_ had fungicidal and bactericidal effects against the *C. albicans* and *E. coli* isolates, demonstrating its antimicrobial potential against clinically relevant resistant microorganisms. This is a common behavior in membrane-targeting antimicrobials, including cationic amino acid-based surfactants [18]. Combined with their amphiphilic nature, this property makes them promising active compounds for use in various pharmaceutical and medical products [26].

### 2.3. C_9_(LA)_2_ Inhibition of Mixed Biofilm Formation of Fluconazole-Resistant C. albicans and ESBL-Producing E. coli

Mixed co-infections caused by Candida and bacteria are more pathogenic than single-species infections. The treatment of mixed fungal and bacterial biofilms poses a significant challenge due to their diverse resistance patterns and dynamic inter-kingdom interactions [27,28]. *E. coli* and *C. albicans* often coexist in human tissues and bodily fluids [29], but studies of new strategies to remove these mixed biofilms are scarce in the literature.

Regarding this, the use of C_9_(LA)_2_ is of great interest because of its activity against resistant bacteria and fungi. Furthermore, due to its significant surface activity and cationic charge located in a guanidine group, it has the potential to modify the surfaces of medical devices or interact with the polymeric matrix of the biofilms through electrostatic and hydrogen bond interactions, facilitating its diffusion through the biofilm.

**Figure 2:**
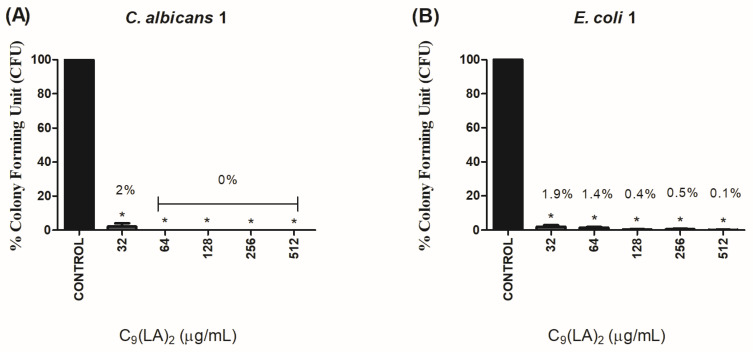
Colony forming unit (CFU) of fluconazole-resistant *C. albicans* (**A**) and ESBL-producing *E. coli* (**B**) mixed biofilms treated with C_9_(LA)_2_. Cells of each specie component of mixed biofilm were quantified and isolated in different culture mediums and expressed with different graphics. Data were submitted to one-way ANOVA followed by the Tukey test. * *p* < 0.05 compared to control.

Therefore, we evaluated the activity of C_9_(LA)_2_ in preventing the formation of mixed biofilms of fluconazole-resistant *C. albicans* and ESBL *E. coli*. The surfactant was able to completely inhibit the formation of mixed biofilms of the tested strains in 96-well plates (Figure 2). This finding corroborates previous reports concerning gemini cationic surfactants as potential antibiofilm molecules acting against a variety of microbial species [30,31].

It can be noted that there was an important reduction in the colony forming unit (CFU) count of both microorganisms (about 98% of CFU reduction, compared to the control) at the lowest concentration evaluated (32 µg/mL). Higher concentrations of C_9_(LA)_2_ showed a % CFU of zero against *C. albicans*, and close to zero at concentrations > 64 µg/mL against *E. coli*. The effectiveness against *E. coli* in mixed biofilms is even better than that observed in planktonic cells. The good antibiofilm activity of this gemini surfactant can be attributed to its important surface activity and its cationic charge situated in a guanidine group.

These results agree with those obtained by Sousa et al. [15]. These authors reported that C_9_(LA)_2_ inhibited the formation of methicillin-resistant *Staphylococcus aureus* (MRSA) ATCC 43300 biofilms by their ability to coat the polystyrene plate through hydrophobic interactions and/or to kill planktonic cells in the solution, which died rapidly upon contact with these surfactants at concentrations equal to or greater than the MIC. Recently, it has also been reported that monocatenary surfactants from arginine and ultrashort lipopeptides containing one or more arginines can effectively eradicate single bacterial biofilms [32,33]. The good eradication of biofilms by these cationic surfactants seems to be due to the destabilization of the extracellular polymeric matrix through electrostatic interactions, dispersing the biofilm and leading to the death of planktonic cells [15,32].

Additionally, Fait et al. [28] described that the high surface activity and positive charge of cationic surfactants can help overcome the *Candida* spp. biofilm matrix by destabilizing its architecture. Due to that, amphiphilic cationic molecules are generally recognized as being able to penetrate the biofilm structure more easily compared to uncharged compounds, thereby exposing sessile cells to the action of the antimicrobial agent.

It has been reported that the interaction between *C. albicans* and *E. coli* is synergistic and can increase mortality [4] due to β-1,3-glucan, a fungal cell wall component also present in the extracellular matrix [34]. Studies reported that in a yeast/*E. coli* dual biofilm, the sensitivity of *C. albicans* to the antifungal nystatin decreased and the MIC against this drug increased from 25 μg/mL to 50 μg/mL [4]. Currently, antimicrobial peptides [35] and biosurfactants [36] are also used to prevent polymicrobial biofilms. Some of these approaches to generating new antibiofilm alternatives require the development of costly synthetic procedures, the use of a mixture of compounds or technical expertise in purifying small molecules or plant extracts. C_9_(LA)_2_ can be an interesting alternative because it can resolve some drawbacks: it does not require the use of more than one compound, it can be prepared using a green economic synthetic procedure and it does not require sophisticated and expensive techniques.

### 2.4. C_9_(LA)_2_ and the Hydrogel Prevented the Formation of Mixed Biofilm of Fluconazole-Resistant C. albicans and ESBL-Producing E. coli in CVCs

Infections induced by CVC are usually associated with bacterial and fungal biofilms, and *C. albicans* along with *E. coli* are often found on medical devices. Hence, the inhibition of catheter biofilm growth is one of the best approaches to preventing the formation of biofilms and consequently reducing these problematic hospital infections [37]. Based on the C_9_(LA)_2_ potential against mixed biofilms of fluconazole-resistant *C. albicans* and ESBL-producing *E. coli* in 96-well plates, we also investigated the preventive potential of this surfactant in silicone-impregnated CVCs due to the clinical relevance of serious infections associated with the colonization of CVCs by these pathogenic microorganisms.

Most of the methods reported in the literature suffer certain limitations. Typically, biofilms are formed on 96-well polystyrene plates, a surface that, while facilitating the optimization of biofilm development, is not clinically relevant. Moreover, studying only reference strains may fail to capture the diversity of resistant strains present in hospital settings. Here, to overcome these limitations, we used a medical device (CVC) and two clinically resistant isolates, a fluconazole-resistant *C. albicans* and an ESBL-producing *E. coli*.

**Figure 3:**
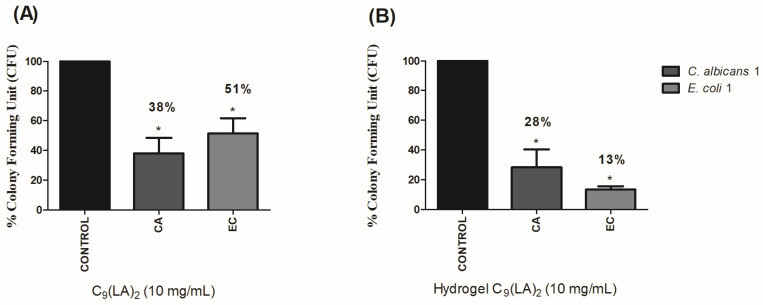
Preventive effect of C_9_(LA)_2_ impregnation (**A**) and its hydrogel (**B**) in CVCs for formation of mixed biofilms of fluconazole-resistant *C. albicans* (CA) and ESBL-producing *E. coli* (EC). Data were submitted to one-way ANOVA followed by Tukey test. * *p* < 0.05 compared to control. CFU: Colony forming unit.

The impregnation of CVCs with C_9_(LA)_2_ significantly (*p* < 0.05) prevented the formation of mixed biofilms in relation to the control, both isolated and loaded in the hydrogel (Figure 3). The hydrogel formulation seemed to improve the antibiofilm activity (Figure 3B). It should be noted that some of the methods reported in the literature require the pre-modification of the substrates, the formation of a bonding layer on the catheter surface to enable covalent bonds between this layer and the hydrogel coating, or introducing surface-active groups that can be covalently grafted to the coating [38]. Here, we used a simple method of preparing the impregnated catheter that does not require complex modifications of the catheter surface.

The good antibiofilm activity of the C_9_(LA)_2_ surfactant can be attributed to its cationic charge and surface properties. It is important to note that the cationic charge of this surfactant is located on the protonated guanidine group, which has a pKa value of approximately 11, and the compound remains protonated even at high pH levels. Thus, these molecules strongly adsorb to the catheter, altering the hydrophobicity of the coated surfaces and blocking the initial stages of biofilm adhesion. In this context, Pontes et al. [39] observed that the presence of sophorolipid biosurfactants on silicone rubber surfaces reduced the hydrophobicity of the material and the formation of mixed biofilms of *S. aureus* and *E. coli*.

Complementary to this, strategies for the use of formulations have been studied within the scope of the antimicrobial activity of gemini arginine-based surfactants. For example, it was found that the gemini surfactant C_3_(CA)_2_ loaded in catanionic vesicles was able to achieve up to a 70% dispersion of the biofilms formed by *P. aeruginosa* ATCC 27853 [40].

The use of hydrogels as coatings for urinary catheters has already been described in the literature. Due to their combination of softness, lubricity and functional network structures that can carry various substances, hydrogels have a wide range of applications in medical devices [38,41]. The CVC impregnated with the gemini-based hydrogel enhanced the effect of the surfactant, as indicated by the greater reduction in CFU values for the hydrogel-impregnated CVC fragments compared to the reduction achieved with the fragments impregnated with C_9_(LA)_2_ alone in relation to their respective controls (Figure 3B). For the impregnation with C_9_(LA)_2_ alone, there were reductions of 62% for *C. albicans* 1 and 48.7% for *E. coli* 1 in terms of CFU values. For the impregnation performed with the surfactant hydrogel, there were decreases of 71.7% for *C. albicans* 1 and 86.7% for *E. coli* 1. There are few reports of new therapeutic alternatives for the prevention of mixed biofilms formed by *C. albicans* and *E. coli*.

The improvement of the antibiofilm activity observed for the gel can be attributed to its three-dimensional network and biological properties. This network structure [42] can provide sufficient capacity to accommodate small antimicrobial molecules that are released at a certain location to achieve antimicrobial effects. Antibiotics are traditional antibacterial agents used in these material coatings to achieve antibacterial properties [43]. Due to the proliferation of resistant bacteria, these drugs can be replaced by other antibacterial substances, such as metal nanoparticles (Ag NPs, Cu NPs or Zn NPs) [44,45,46]. However, it has been reported that some of those molecules loaded in the hydrogels (Ag^+^ or Cu^2+^) exhibited toxicity against mammalian cells [47], which limits their application in the biomedical field.

In this context, hydrogels containing amino-acid-based surfactants with antimicrobial activity, such as C_9_(LA)_2_, are an interesting alternative due to their biological, physicochemical and environmental properties, as well as their straightforward preparation methods. To date, this is the first study describing the use of hydrogels containing amino-acid-based surfactants as coatings for CVCs to inhibit the formation of mixed *C. albicans/E. coli* biofilms. Only Silva et al. [14] have reported the use of a gel containing a cationic biosurfactant complexed to arginine to coat a peripheral venous catheter and observed the enhancement of prevention caused by gel formulations against fluconazole-resistant *C. albicans* and MRSA mixed biofilm.

In principle, the development of resistance to these hydrogels by bacteria and fungi is unlikely. On the one hand, the mode of action of this compound seems to involve the bacterial or fungal membrane disruption. On the other hand, this compound is biodegradable [16], which prevents the appearance of resistant strains due to prolonged exposure of microorganisms to concentrations above their MICs [48].

### 2.5. Effect of C_9_(LA)_2_ and Hydrogel Against Fungal and Bacterial Ultrastructure’s

**Figure 4:**
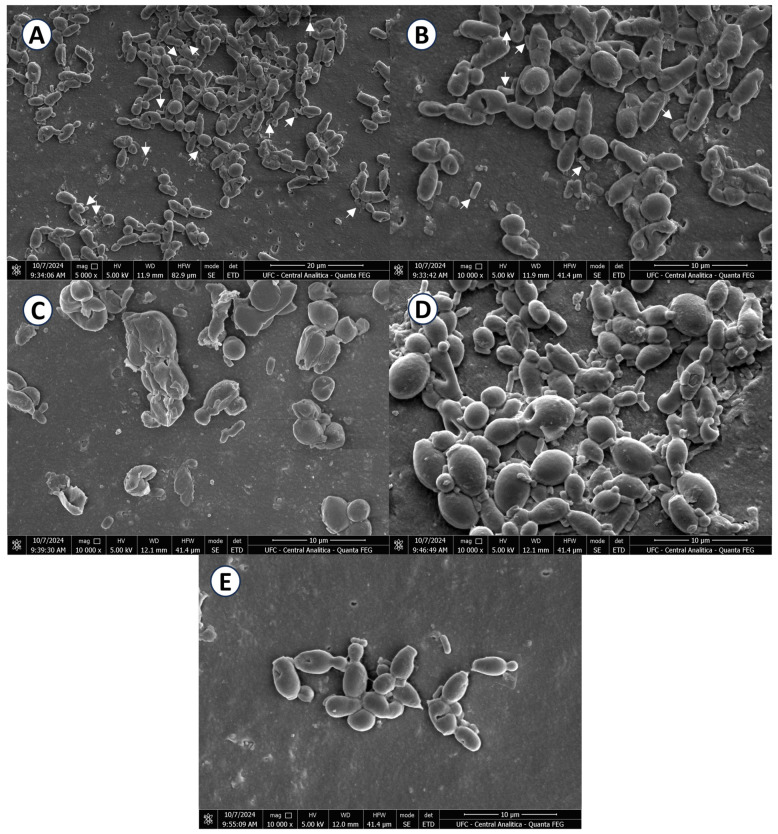
Preventive effect of C_9_(LA)_2_ and its hydrogel impregnation of CVC fragments evidenced by SEM in formation of mixed biofilm of fluconazole-resistant *C. albicans* and ESBL-producing *E. coli*. (**A**,**B**) Control with different magnification; *E. coli* cells are indicated by white arrows. (**C**) C_9_(LA)_2_ impregnation. (**D**) Hydrogel blank (without C_9_(LA)_2_) impregnation. (**E**) Impregnation of hydrogel (containing C_9_(LA)_2_). (**A**) Magnification: 5000×, Bar: 20 µm. (**B**–**E**) Magnification: 10,000×. Bar: 10 µm.

The microstructure of the mixed biofilm formed by fluconazole-resistant *C. albicans* and ESBL-producing *E. coli* in the CVC was visualized by SEM (Figure 4). SEM is one of the most widely used techniques for the qualitative structural characterization of biofilms due to its high resolution [49]. A dense polymicrobial biofilm with clusters of intact fungal and bacterial cells was observed in the CVC fragments subjected to impregnation only in chloroform (Figure 4A) and in the CVC fragments impregnated with the hydrogel blank (Figure 4C). In the CVCs impregnated with C_9_(LA)_2_ (Figure 4B), a reduction in the number of microbial cells with cellular debris and the presence of cell damage were observed. The CVC fragments impregnated with the hydrogel containing the surfactant (Figure 4D) showed a drastic reduction in cell density. The CVC images obtained by SEM corroborated the data acquired from colony counts of catheters. Similar morphological damages were observed by Ashrit et al. [50] and Esfandiary et al. [51] against *C. albicans*/*E. coli* mixed biofilms treated with a garlic extract and oleuropein, respectively.

### 2.6. C_9_(LA)_2_ Did Not Induce Cytotoxicity in Human Peripheral Blood Lymphocyte (PBL) Cells

Good biocompatibility is an important requirement for all materials used in biomedical applications. In order to determine the biocompatibility of these surfactants, we examined the effects of C_9_(LA)_2_ on the viability of cultured PBLs. PBLs are mature lymphocytes that circulate in the bloodstream rather than residing in specific organs. They are one of several types of white blood cells that play a crucial role in the immune system [52]. These mammalian cells were treated with the C_9_(LA)_2_ and the colorimetric 3-[4,5-dimethylthiazol-2-yl]-2,5 diphenyl tetrazolium bromide assay (MTT) was used to examine the cytotoxicity of this surfactant in vitro.

The cytotoxicity of this surfactant was measured at different concentrations. The maximum concentration tested was 500 µg/mL, a concentration well above the obtained MIC. We observed that this compound did not suppress the proliferation of PBL cells after incubation for 24 h at any of the tested concentrations. This indicates that C_9_(LA)_2_ has a very high IC_50_ value (>500 µg/mL) (the concentration of surfactant that inhibits 50% of the cell proliferation). Notably, the MICs obtained for all tested microbes were below the IC_50_, demonstrating the good selectivity of this surfactant against bacteria and fungi, as well as a very high therapeutic index (TI = IC_50_/MIC) for all of the tested *Candida* species. Considering the MICs, the surfactant demonstrated a TI > 185 for *Candida* and TI > 1,6 for *E. coli*. Additionally, it has been reported that this compound exhibits low hemolytic activity. The low cytotoxicity of this surfactant was attributed to the large aggregates formed in its aqueous solution.

## 3. Materials and Methods

### 3.1. Fungal and Bacterial Isolates

We used eight clinical isolates: four fluconazole-resistant *C. albicans* and four ESBL-producing *E. coli*. In addition, we included *C. parapsilosis* ATCC 22019, *C. krusei* ATCC 6258, *E. coli* ATCC 8739 and *E. coli* ATCC 25922 as control strains. All isolates belong to the collection of microorganisms of the Laboratory for Bioprospection of Antimicrobial Molecules (LABIMAN) of Federal University of Ceará (UFC).

### 3.2. Surfactant and Hydrogel Production

The surfactant C_9_(LA)_2_ was designed and synthesized by the Biocompatible Surfactants and Ionic Liquids Research Group (IQAC/CSIC). The synthesis protocol was performed according to Pérez et al. [17]. Briefly, it was obtainedwith a purity of 99% via chemical condensation of the single N^α^-lauroyl-L-arginine to the corresponding 1,9 diaminononane in the presence of an activating agent. A final deprotection reaction was carried out to obtain the pure compound. NMR spectroscopy, mass spectroscopy and HPLC characterization results (Figure 1) agreed with the those reported by [17].

Furthermore, a hydrogel was produced with C_9_(LA)_2_ at the Polymer and Materials Innovation Laboratory (LabPIM) of UFC. The hydrogel was prepared based on preliminary solubility and stability tests with different hydrophilic polymers. PEG 600 (Sigma-Aldrich, St. Louis, MO, USA) produced the best results in terms of solubility and stability of the arginine gemini-based surfactant C_9_(LA)_2_, and it was chosen as the basic component of the gel matrix. The components were added based on their percentage of the final mass of the formulation. Initially, the surfactant C_9_(LA)_2_ was weighed to achieve a concentration of 20 mg/mL (2% *w*/*w*) and then was mixed with dimethyl sulfoxide (DMSO) (NEON Comercial, Suzano, SP, Brazil) at a concentration of 2.5% *w*/*w* until it reached complete solubilization. Next, PEG 600 was added at a concentration of 40% *w*/*w* and stirred using an IKA MS 3 Basic shaker (Wilmington, DE, USA) at 2500 rpm for 15 min. Simultaneously, an aqueous solution of Pluronic F-127 (Sigma-Aldrich, St. Louis, MO, USA) was prepared at 2% *w*/*w*, cooled until completely dissolved and then slowly added to the PEG-C_9_(LA)_2_ solution via dripping with gentle stirring using an IKA MS 3 Basic shaker at 500 rpm until the formulation was fully homogenized.

### 3.3. Antimicrobial Susceptibility Testing (AST) and Breakpoints

For fungal and bacterial isolates, antimicrobial susceptibility testing (AST) was conducted using broth microdilution assay. Antifungal and antibacterial activity of C_9_(LA)_2_ were assessed according to CLSI broth microdilution protocols M27-A3 [53] and M07-A10 [54], and MICs were considered the lower concentrations that completely inhibited microbial growth. The reading of MICs was performed using a microplate reader (SmartreaderTM 96, Accuris Instruments, Edison, NJ, USA) at 450 nm [55], standardized for fungal and bacterial isolates in order to provide more sensitive MIC determination.

The resistance breakpoints of the isolates were previously verified [14,56]. For fluconazole, these were analyzed according to CLSI document M27-S4 [57] for *Candida* spp., whereby MIC ≥ 8 µg/mL indicates resistance of the clinical isolates of *C. albicans* used in the study. Following the guidelines described in the M27-A3 document [53], we used a fluconazole-resistant standard strain (*C. krusei* ATCC 6258) and a fluconazole-sensitive strain (*C. parapsilosis* ATCC 22019) as references. The MIC values obtained for fluconazole (reference antifungal compound) were 1 µg/mL for *C. parapsilosis* ATCC 22019 and 16 µg/mL for *C. krusei* ATCC 6258, consistent with previous results reported by Silva et al. [14]. These MIC values align with those reported for this compound in the CLSI document M27-S4 [57]. Notably, in accordance with the CLSI M27-A3 document [53], the endpoint for fluconazole MIC determination was defined as the lowest concentration at which prominent growth inhibition was observed (≥50% inhibition compared to the growth control well).

For *E. coli* strains, ESBL detection was previously performed according to the EUCAST disk combination test [58]. Moreover, ciprofloxacin resistance breakpoints for *E. coli* were verified according to CLSI document M100-S31 [59], whereby MIC ≥ 1 µg/mL indicates resistance of the ESBL-producing *E. coli* clinical isolates used in this study.

### 3.4. MFC and MBC

After reading the broth microdilution plates, aliquots (10 µL) were taken from the wells corresponding to MIC, 2xMIC and 4xMIC, and were subsequently plated on potato dextrose agar (PDA) (Kasvi, Pinhais, PR, Brazil) for *Candida* spp. isolates and brain heart infusion (BHI) agar (HiMedia, Mumbai, MH, India) for *E. coli* isolates. After 24 h incubation at 35 °C, the lowest concentration at which no microbial growth was observed on the surface of the agar medium was determined as the MFC (fungi) or MBC (bacteria) [56,60].

From this, the ratios of MFC and MBC with the MIC were calculated to determine the tolerance level, where a value ≤4 indicates that the drug has fungicidal/bactericidal action, and a value >4 indicates fungistatic/bacteriostatic action [61,62].

### 3.5. Analysis of the Action of C_9_(LA)_2_ Against Mixed Biofilm Formation of Fluconazole-Resistant C. albicans and ESBL-Producing E. coli

The protocol was executed according to Eshima et al. [63] and Silva et al. [56], with some modifications. Initially, 24 h cultures of the representative isolates *C. albicans* 1 and *E. coli* 1 were prepared in yeast nitrogen base (YNB) medium (Difco, Becton Dickinson Sparks, MD, USA) supplemented with 100 mM of glucose (Isofar, Duque de Caxias, RJ, Brazil) and tryptic soy broth (TSB) (Kasvi, Pinhais, PR, Brazil) supplemented with 2% glucose, respectively. The cultures were then centrifuged (2500 rpm for 5 min) and washed with saline solution (0.85% NaCl) three times. Immediately afterward, inoculum was prepared in BHI broth (HiMedia, Mumbai, MH, India) supplemented with 2% glucose. For *C. albicans*, the 0.5 McFarland scale (1 × 10^6^ CFU/mL) was used as the standard, while for *E. coli*, the McFarland scale 1 (3 × 10^8^) was used. In a 96-well flat-bottom plate, 50 µL of the inoculum corresponding to *C. albicans*, 50 µL of the inoculum corresponding to *E. coli* and 100 µL of the C_9_(LA)_2_ dilutions were added to each well, followed by incubation for 48 h at 35 °C. The surfactant was evaluated in a standardized concentration range between 32 and 512 µg/mL. Wells were designated to monitor the sterility of the culture medium and to assess the growth of microorganisms.

After washing the wells to remove non-adherent cells, 100 µL of saline solution was added, followed by the detachment of the biofilm. From this, two consecutive dilutions were performed in the proportions of 10 µL and 990 µL of saline solution, followed by seeding on Sabouraud dextrose agar (SDA) (Kasvi, Pinhais, PR, Brazil) supplemented with chloramphenicol (Sigma-Aldrich, St. Louis, MO, USA) and tryptic soy agar (TSA) (HiMedia, Mumbai, MH, India) supplemented with amphotericin B (Sigma-Aldrich, St. Louis, MO, USA) [64] in order to perform a selective CFU count for *C. albicans* and *E. coli*, respectively. The plates were incubated for 24 h at 35 °C, and the results were analyzed as the percentage of CFUs of the treatments compared to the respective microbial growth controls. The assays were executed in 2 independent experiments with 5 replicates each one.

### 3.6. Impregnation of C_9_(LA)_2_ and Its Hydrogel Formulation in CVC Fragments

The procedure was performed according to Fisher et al. [65] and Nair et al. [66]. Briefly, 0.5 cm fragments of silicone CVC (Blenta, Indaiatuba, SP, Brazil) were impregnated with the surfactant and its respective hydrogel at a concentration of 10 mg/mL (1% *w*/*v*) of C_9_(LA)_2_. This concentration for impregnation was based on previous studies that used a value in the range of % *w*/*v*, as described by Fisher et al. [65] and Silva et al. [14], which used variations from 0.2 to 1%.

Thus, for C_9_(LA)_2_ impregnation, catheter fragments were submerged in chloroform with the surfactant at a concentration of 10 mg/mL for 1 h at 35 °C. In addition, catheter fragments submerged in chloroform (Dinâmica, Indaiatuba, SP, Brazil) without C_9_(LA)_2_ were subjected to the same conditions for subsequent control of microbial growth. Similar to hydrogel impregnation, catheter fragments were submerged in chloroform for 1 h at 35 °C, followed by a new incubation with the hydrogel at 10 mg/mL for 1 h at 60 °C, a temperature at which the stability and fluidity were enhanced. Catheter fragments were also impregnated with the blank hydrogel (without C_9_(LA)_2_) as a control. Finally, the catheter fragments were transferred to sterile Petri dishes and placed to dry in an oven for 24 h at 35 °C [14]. The assays were executed in 2 independent experiments with 5 replicates each one.

### 3.7. Determination of the Potential of C_9_(LA)_2_ and Its Hydrogel Formulation to Prevent Mixed Biofilm Formation of Fluconazole-Resistant C. albicans and ESBL-Producing E. coli in CVC Fragments

In a 24-well plate, catheter fragments were transferred according to treatments and controls. The inoculum for the assay was prepared as previously described, using the same representative isolates. Then, 250 µL of fungal inoculum and 250 µL of bacterial inoculum were added, and the plate was incubated for 48 h at 35 °C. After this period, the catheter fragments were gently washed and transferred to a test tube containing 1 mL of saline solution, which was vortexed for 1.5 min to ensure that the microbial cells adhered to the catheter were released into the solution [14]. Then, 10 µL was seeded in SDA supplemented with chloramphenicol and TSA supplemented with amphotericin B [64]. The plates were incubated for 48 h at 35 °C, and CFUs were subsequently counted and converted into a percentage compared to microbial growth controls.

### 3.8. Data Analysis

Independent replicates were performed for the assays. To evaluate significant differences, the data were submitted to one-way analysis of variance (ANOVA) followed by the Tukey test (*p* < 0.05). The data were analyzed using GraphPad Prism version 8.0.1 (GraphPad Software, San Diego, CA, USA).

### 3.9. SEM Evaluation

SEM was performed on catheter fragments submerged only in chloroform (without C_9_(LA)_2_), as well as on fragments impregnated with C_9_(LA)_2_, its hydrogel and hydrogel blank, all subjected to the conditions of mixed biofilm formation of *C. albicans* 1 and *E. coli* 1, used as representatives for the tests.

The catheter fragments were impregnated as previously described. The inoculum preparation was also performed under the same conditions previously described. Thus, the catheter fragments were transferred to a 12-well plate, to which 250 µL of fungal inoculum and 250 µL of bacterial inoculum were added, and the plate was incubated for 48 h at 35 °C. Then, the catheter fragments were gently washed and a fixation solution containing 2.5% glutaraldehyde (Sigma-Aldrich Co., St. Louis, MO, USA) in 0.15 M cacodylate buffer (Electron Microscopy Sciences, Hatfield, PA, USA) and 0.1% Alcian blue (Sigma-Aldrich Co., St. Louis, MO, USA) was added, followed by overnight incubation at 4 °C. Afterwards, the catheter fragments were washed twice with a 0.15 M cacodylate buffer solution, and then, the alcoholic dehydration process was performed. Afterwards, the catheter fragments were placed in an oven at 35 °C to dry and were subsequently dehydrated with hexamethyldisilazane (Sigma-Aldrich Co., St. Louis, MO, USA) [56]. The samples were coated with a 20 nm layer of gold (Emitech, Uckfield, UK; Q150T) and the analyses were performed using a FEI Quanta 450 FEG microscope (FEI Company, Waltham, MA, USA) at the UFC Analytical Center.

### 3.10. Evaluation of C_9_(LA)_2_ Cytotoxicity

#### 3.10.1. Isolation of PBLs

Cells were isolated by Histopaque-1077 density gradient centrifugation [67]. Samples were collected from healthy patients. The protocol was approved by the Human Research Ethics Committee of Federal University of Ceará (protocol number 161/2014). Briefly, cells were washed and resuspended in Roswell Park Memorial Institute (RPMI) 1640 medium supplemented with fetal bovine serum (20%), glutamine (2 mM), penicillin (100 U/mL) and streptomycin (100 μg/mL), at 37 °C under 5% CO_2_. Phytohemagglutinin (2.5%) was added at the beginning of the culture.

#### 3.10.2. Assessment of Cell Viability of PBLs

Viability was quantified by the 3-(4,5-dimethyl-2-thiozolyl)-2,5-diphenyl-2H-tetrazolium bromide (MTT) reduction assay (Sigma-Aldrich, St. Louis, MO, USA) [68]. For the tests, cells were seeded in 96-well plates (1.5 × 10^6^ cells/mL). After 48 h, C_9_(LA)_2_ (0.97 to 500 μg/mL) was added to the wells and the cells were incubated for 24 h. Subsequently, the plates were centrifuged and the medium was replaced, with the addition of 150 μL of MTT (0.5 mg/mL) and incubation for 3 h. After this period, 150 μL of DMSO (Dinâmica, Indaiatuba, São Paulo, Brazil) was added and the absorbance was measured using a multiplate reader (Spectra Count, Packard, Windsor, ON, Canada) at 595 nm.

## 4. Conclusions

We investigated the antimicrobial activity of a gemini surfactant based on arginine, C_9_(LA)_2_, against clinically resistant isolates of *C. albicans* and *E. coli*. This compound showed very good efficiency in killing resistant *C. albicans* strains and moderate activity against resistant *E. coli* strains. Interestingly, this compound was biocompatible with PBL cells at higher concentrations than its MIC values. Additionally, the results indicate that impregnating CVCs with the gemini cationic surfactant C9(LA)2 can modify the hydrophobic properties of the catheter surface, effectively inhibiting the development of mixed *C. albicans*/*E. coli* biofilms. Notably, an enhanced antibiofilm effect was observed when the CVC fragments were impregnated with a hydrogel containing the gemini surfactant. These findings suggest that both the surfactant alone and its hydrogel formulation could serve as effective strategies for preventing biofilm formation on medical devices, thereby potentially reducing the incidence of catheter-associated infections.

This work represents a first step towards the exploration of cationic gemini surfactants’ ability to inhibit interkingdom biofilm growth on medical devices. The results indicate that this kind of surfactant can be an interesting candidate for these medical applications. It should be noted that due to its mode of action and biodegradability, the development of resistant microorganisms to the proposed antibiofilm substance is very unlikely.

## Figures and Tables

**Table 1:** The antifungal effect of C_9_(LA)_2_ against *Candida* spp. strains.

Strains ^a^	MIC ^b^	MFC ^c^	Tolerance Level ^d^	Interpretation ^e^
*C. parapsilosis* ATCC 22019	4	5.3	1.3	Fungicide
*C. krusei* ATCC 6258	6.7	6.7	1	Fungicide
*C. albicans* 1 *	5.3	6.7	1.3	Fungicide
*C. albicans* 2	5.3	5.3	1	Fungicide
*C. albicans* 3	5.3	5.3	1	Fungicide
*C. albicans* 4	4	6.7	1.7	Fungicide

^a^ *Candida* spp. strains belong to the arsenal of isolates of the Laboratory for Bioprospection of Antimicrobial Molecules (LABIMAN). All clinical *C. albicans* isolates are resistant to fluconazole. ^b^ The lowest concentration that completely inhibited fungal growth after the incubation period (µg/mL). ^c^ The minimum fungicidal concentration (µg/mL). ^d^ The MFC/MIC ratio. ^e^ A tolerance level < 4 is interpreted as fungicidal action. * The representative isolate used in plaque and catheter biofilm assays and scanning electron microscopy (SEM).

**Table 2:** The antibacterial effect of C_9_(LA)_2_ against *E. coli* strains.

Strains ^a^	MIC ^b^	MBC ^c^	Tolerance Level ^d^	Interpretation ^e^
*E. coli* ATCC 8739	85.3	106.7	1.2	Bactericide
*E. coli* ATCC 25922	128	128	1	Bactericide
*E. coli* 1 *	298.7	341.3	1.1	Bactericide
*E. coli* 2	128	128	1	Bactericide
*E. coli* 3	170.7	170.7	1	Bactericide
*E. coli* 4	170.7	170.7	1	Bactericide

^a^ *E. coli* strains belong to the arsenal of isolates of the Laboratory for Bioprospection of Antimicrobial Molecules (LABIMAN). All clinical *E. coli* isolates are ESBL. ^b^ The lowest concentration that completely inhibited bacterial growth after the incubation period (µg/mL). ^c^ The minimum bactericidal concentration (µg/mL). ^d^ The MBC/MIC ratio. ^e^ A tolerance level <4 is interpreted as bactericidal action. * The representative isolate used in plaque and catheter biofilm assays and SEM.

## Data Availability

The original contributions and essential data supporting the reported results presented in this study are included in the article. Further inquiries can be directed at the corresponding authors.
